# PRODUCT REVIEW / ÉVALUATION DE PRODUIT

**DOI:** 10.29173/jchla29854

**Published:** 2025-08-01

**Authors:** Dean Giustini

**Affiliations:** UBC Biomedical Branch Librarian Liaison librarian: UBC Faculty of Medicine, St. Paul's and Vancouver hospitals VGH Diamond Health Care Centre, UBC Library Vancouver, BC, Canada

**Product:** Undermind.ai

**URL:** https://www.undermind.ai/

## Product description/purpose

Undermind.ai is a “next generation, AI-powered information retrieval system that researches a complex topic for you” [1]. The tool mimics a human searcher’s multi-step discovery process and adapts its searching dynamically by leveraging artificial intelligence (AI). In 2023, as a Silicon Valley start-up, Undermind was described as “Google for scientific research” [2], its founders saying: “as researchers ourselves [we wanted to build] a search engine that could handle extremely complex questions… geared at experts, like research scientists and doctors, who need to find very specific resources to solve high-stakes problems” [2].

Undermind.ai performs searches in Semantic Scholar (https://www.semanticscholar.org), an interdisciplinary database of 225 million citations [3], via an application programming interface (API)–paired with a large language model (LLM)–to process results [4]. Instead of executing a single search, “Undermind’s algorithm conducts multiple iterative searches, dynamically adjusting its approach based on previously retrieved results, and carefully reading and following citation trails” [5].

## Intended audience/users

Undermind is geared towards scientists and researchers with expert level information needs [6]. According to Shaurya Pednekar, an engineer at Undermind, “many users are research scientists at pharmaceutical and biotech companies in addition to academics” [5]. Librarians working with faculty, and upper level undergraduate and graduate students may find it useful in teaching AI search technologies, but probably not as a starter tool. Undermind is not suitable for clinicians needing quick answers at point-of-care, or for those doing quick Googling.

## Special features

**Fig. 1:**
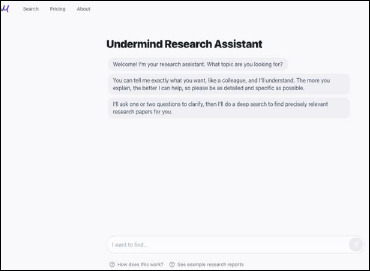
Undermind’s new search homepage (https://app.undermind.ai/) as of March 12th, 2025 where searchers engage with the AI assistant

**Fig. 2:**
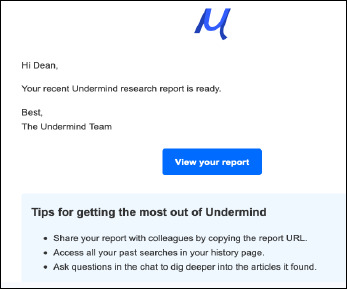
An emailed report sent after Undermind completes your search

Here are a few notable features of Undermind.ai:

1. *AI Agent*: The AI agent acts like a chatbot, interacting with researchers initially to refine their research question by asking “What do you want to know?” and “Tell me exactly what you want, like a colleague” (Figure 1). This feature is reminiscent of what librarians do in their consultations and reference interviews.
2. *Deep Search Technology*: Deep Search goes beyond keyword searching to the document’s meaning (semantics), performing multiple, successive searches based on what the system finds in relevant papers; Undermind “leverages the latest AI using neural nets to allow matching of queries to documents” [6].
3. *LLM-Classifiers and Summaries*: Large language models are used as “a reasoning engine and classifier at key steps within a structured search process” [7], generating summaries to help researchers understand the retrieved corpus and presenting papers in tables with match scores for scanning and analysis. Undermind uses GPT-4, an LLM that is part of the GPT (Generative Pre trained Transformer) series at OpenAI [4].
4. *Organized Email Report*: Undermind sends a report of papers it found via email in 8-10 minutes (Figure 2). The report reveals how many relevant papers were found (estimating, for example, that it located 93% of relevant papers after analyzing 144), matches scores out of 100 for each paper, and provides a citation network and timeline. Influential papers are at the top, less cited ones lower down. Using generative AI, the “Write a short review article” feature will compose an evidence based narrative based on the papers. New queries can also be generated at this point.

## Example searches

**Fig. 3:**
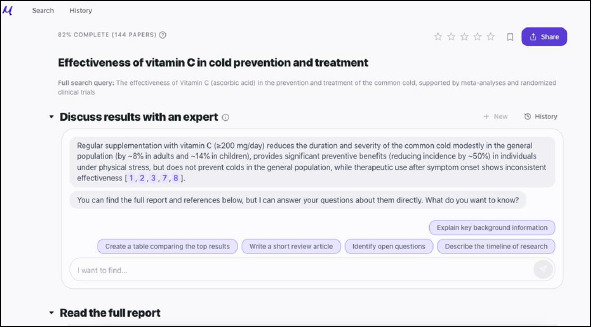
Undermind’s response to a basic search query in biomedicine, “Is vitamin c effective in treating common cold?–find meta-analyses and randomized clinical trials”

**Fig. 4:**
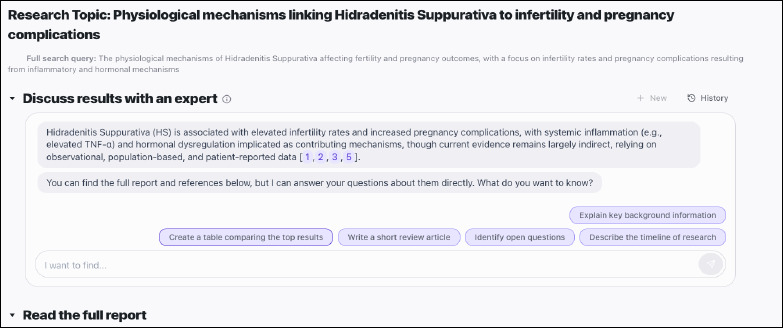
Undermind’s response to an advanced research query in biomedicine, “What are the physiological mechanisms through which Hidradenitis Suppurativa impacts fertility and pregnancy outcomes, particularly regarding infertility rates and pregnancy complications driven by inflammatory and hormonal factors?”

Here are two examples of searches in Undermind —a basic search on the effectiveness of treating a common cold and an advanced search on a complex biomedical question:

1. Basic Search query (Figure 3): “Is Vitamin C (ascorbic acid) effective in treating common cold?” (a basic report is available, and can be viewed here: https://app.undermind.ai/report/e61db6812482cc1248dd13fa4dc2b7652f2ce589c0e5d748a8e456e880fcb7b8) .
  According to Josh Ramette at Undermind: “...some research questions (such as “vitamin C and common cold”) may be better served at Google Scholar or other search tool. To take full advantage of the power of Undermind, really complex, cutting edge research topics are more appropriate based on the complexity of the system” [8].
2. Advanced search query (Figure 4): “What are the physiological mechanisms through which Hidradenitis Suppurativa impacts fertility and pregnancy outcomes, particularly regarding infertility rates and pregnancy complications driven by inflammatory and hormonal factors?” (A basic report is available, and can be viewed here: https://app.undermind.ai/report/a0f894bdcf1592dd72311d3d78d6d4e3b8e0300b48be26d2b02a06e74f753652).

## Compatibility issues

Undermind requires no local software installation, or downloads. Signing up for a free account at the website is recommended, and a reliable Internet connection is essential. Pro users can export results directly into a citation manager (e.g., Zotero, EndNote) or screening platform (e.g., Covidence) using a standard file format (e.g., CSV, BibTeX and RIS). Evidence based clinicians would benefit from a simpler mobile version, given the interface’s complexity. Browser compatibility is acceptable in Chrome, Firefox, and Edge.

## Platform

No downloadable client is needed. Searches are saved on the platform, with shareable links. The interface has a minimal feel initially but gets cluttered quickly. Librarians accustomed to thesaurus based Boolean searching may find the user interface inflexible, but novices may prefer the platform’s ability to search and synthesize papers. Authentication is via username and password. The “Terms of Use” states that you can “freely share, distribute, and disseminate search results…with any third parties, including but not limited to non subscribers, other institutions, and the general public” [9].

## Usability

Undermind relies on the user to communicate their information needs to the research assistant. The system mitigates this by asking users to clarify their research question, and reframe it in several steps. The 8-10 minute wait may frustrate those expecting faster results, though the relevance scores and “Summary of Key Findings” in the emailed report are informative. It is unclear whether searchers will find the detailed relevancy tables helpful, but they will provide some utility and transparency. Documentation is sparse for this tool, with only one video and the site FAQs. Finally, editing searches is not possible, but searchers can return to reports via history to ask new questions of the system, which increases the usability of search histories.

## Strengths and weaknesses

Undermind’s use of an AI agent or chatbot to refine a query is a strength, especially for complex topics. Another is the system’s statistical model or “discovery curve” that estimates the total number of relevant papers on any given scientific topic–even for cutting edge topics where there may only be a handful of relevant papers. Aaron Tay, librarian at Singapore Management University, said the model is based on the idea that “when users start to exhaust relevant papers in an index, they will start getting fewer and fewer relevant papers. This feature offers users confidence that they aren't missing highly relevant content” [6].

The inability to report a reproducible search strategy is Undermind’s Achilles heel, but most AI search platforms have this limitation. The cognitive load to learn an AI based user interface and system is high, but manageable via trial and error testing. Given the algorithms in this product, librarians will want to evaluate whether their search results reveal or perpetuate biases of various kinds [10]. The use of OpenAI GPT-4 may raise ethical questions about how to ethically use AI tools, and librarians should be prepared to have those conversations [11]. Due to the computationally intensive nature of the system, the platform prioritizes precision over speed. Some researchers will find the wait for results to be too long.

## Comparison with similar products

- Google Scholar (GS) (https://scholar.google.com) is the largest interdisciplinary search engine with ~400 million citations and used by researchers, despite the browsing and iterative searching required. What Undermind lacks in GS’ breadth of coverage and speed, it makes up for with its unique algorithms and syntheses.
- PubMed.gov (http://www.pubmed.gov) is the National Library of Medicine (NLM)’s free MEDLINE search index to 37 million citations and used to access structured search features and Medical Subject Headings (MeSH). Undermind.ai offers synthesis but no MeSH integration. The search strategies are also not reportable, transparent, or reproducible.
- Elicit.com (https://elicit.com) integrates generative AI and LLMs, and now offers a “Start A Systematic Review” workflow to support researchers in knowledge synthesis projects. (Undermind’s iterative algorithms and depth may edge out Elicit for complex queries).
- SciSpace (https://scispace.com) “AI Chat for Scientific Research” focuses on visualization and summaries. Less iterative than Undermind, SciSpace is more suitable for quick insights than a deeper synthesis, though it recently released its Deep Review feature: https://scispace.com/search.

## Currency

Undermind.ai uses Semantic Scholar’s updated database in real time, ensuring its currency. The platform’s AI driven search process enhances its currency by adaptively exploring full text and citation networks, similar to a human researcher’s process. Undermind continues to evolve—the 2023 version searched arXiv.org only but it now includes Semantic Scholar’s content from PubMed, arXiv, and other databases. If there is a time lag, it is likely a day or two only. The use of GPT-4 and Deep Search Technology suggest the system is current.

## Cost/value

Undermind.ai offers a free version with a five search limit per month with each search analyzing 50 papers. The subscription pro version analyzes more than 150 papers. The pro version pricing is based on usage or simultaneous users (e.g., $60USD per month billed annually per user), and includes pricing for “Teams” and “Enterprise”. For librarians, the return on investment will depend on the time saved in synthesizing papers. Compared to free tools such as Google Scholar or subscription databases (Ovid MEDLINE), I would call this a boutique item for high end researchers, but not appropriate for general reference or undergraduate students unless they are interested in AI enabled searches.

## Contact information

- Website: https://www.undermind.ai/home/
- For support, communicate with staff via Undermind.ai website. o Direct email: (support@undermind.ai)
- Community: Limited public presence—social media via X (https://x.com/UndermindAI) and LinkedIn (https://www.linkedin.com/company/undermind-ai).

## Conclusion

Undermind.ai is a worthy, niche competitor in the AI search space, and researchers will benefit from using it. For health sciences librarians, it offers an AI powered way to start a literature review in biomedicine, and to find highly relevant (seed) papers in support of knowledge syntheses. The system’s slow response time limits its utility in some contexts, despite helpful summaries, match scores, and a final report. Similar to Elicit.com, Undermind is a sophisticated option for researchers, and represents the future direction of AI search tools [12]. Users should weigh buying a subscription and the platform’s shortcomings against the time savings offered by the final report.

## References

[1] Undermind. [Homepage] [Internet]. [place unknown]: Undermind AI, Inc.; c2025 [cited 2025 Apr 22]. Available from: https://www.undermind.ai/.

[2] Y-Combinator [Internet]. [place unknown]: Y Combinator; c2025. Undermind: An AI agent for scientific research [date unknown] [cited 2025 Mar 5]; [about 5 p.] Available from: https://www.ycombinator.com/companies/undermind.

[3] Semantic Scholar [Internet]. Seattle (WA): Ai2; [date unknown]. About Semantic Scholar; [date unknown] [cited 2025 Mar 5]; [about 4 p.]. Available from: https://www.semanticscholar.org/about.

[4] Ramette, J. Discussion of Undermind.ai [Internet]. Zoom transcript. Discussion with D. Giustini. 2025 Feb 3 [cited 2025 Mar 5]. [3 paragraphs].

[5] Pednekar, S. Discussion of Undermind.ai [Internet]. Email message to: D. Giustini. 2025 Jan 30 [cited 2025 Mar 5].

[6] Tay A. New AI tool shows the power of successive search. Katina [Internet]. 2024 Nov 12 [cited 2025 Mar 5];[about 12 p.]Available from: https://katinamagazine.org/content/article/main-section/2024/undermind-ai-shows-the-power-of-successive-search.

[7] Hartke T, Ramette J. Benchmarking the Undermind Search Assistant [Internet]. 2024 [place unknown]: Undermind AI Inc.; Jan 5. 11 p. [cited 2025 Mar 5]. Available from: https://www.undermind.ai/static/Undermind_whitepaper.pdf.

[8] Ramette, J. Discussion of Undermind.ai [Internet]. Message to: D. Giustini. 2025 Jan 30 [cited 2025 Mar 5]. [3 paragraphs].

[9] Undermind [Internet]. [place unknown]: Undermind AI, Inc.; c2025. Terms of use; 2024 Dec 6 [cited 2025 Mar 5]; [about 14 p.] Available from: https://www.undermind.ai/terms_of_use/.

[10] Saeidnia HR. Ethical artificial intelligence (AI): confronting bias and discrimination in the library and information industry. Library Hi Tech News [Internet]. 2023 Oct 24. [cited 2025 Mar 5]. Available from: https://www.emerald.com/insight/content/doi/10.1108/lhtn-10-2023-0182/full/html.

[11] Ngulube P, Vincent Mosha NF. Integrating artificial intelligence-based technologies ‘safely’ in academic libraries: An overview through a scoping review. Technical Services Quarterly. 2025 Jan 2;42(1):46-67.

[12] Fortier-Dubois E. How we evaluated Elicit Reports. 2025 Mar 4 [cited 2025 Mar 5]. In: Elicit Blog [Internet]. [place unknown]: Elicit. c2025. [about 5 p.]. Available from: https://blog.elicit.com/elicit-reports-eval/.

